# Structural characteristics, functions, and counteracting strategies of biofilms in *Staphylococcus aureus*

**DOI:** 10.1016/j.csbj.2025.01.021

**Published:** 2025-01-23

**Authors:** Yanze Xia, Zhenghui Hu, Qiyuan Jin, Qi Chen, Chenhao Zhao, Rui Qiang, Zonggang Xie, Liubing Li, Haifang Zhang

**Affiliations:** a Department of Orthopedics, The Second Affiliated Hospital of Soochow University, Suzhou, China; b Department of Clinical Laboratory, The Second Affiliated Hospital of Soochow University, Suzhou, Jiangsu, China; c MOE Key Laboratory of Geriatric Diseases and Immunology, Soochow University, Suzhou, China

**Keywords:** *Staphylococcus aureus*, Biofilm, Drug resistance, Innovative therapeutic strategies

## Abstract

**Background:**

*Staphylococcus aureus* (*S. aureus*) is a prevalent pathogen associated with a wide range of infections, exhibiting significant antibiotic resistance and posing therapeutic challenges in clinical settings. The formation of biofilms contributes to the emergence of resistant strains, further diminishing the efficacy of antibiotics. This, in turn, leads to chronic and recurrent infections, ultimately increasing the healthcare burden. Consequently, preventing and eliminating biofilms has become a critical focus in clinical management and research.

**Aim of review:**

This review systematically examines the mechanisms underlying biofilm formation in *S. aureus* and its contribution to antibiotic resistance, emphasizing the essential roles biofilms play in maintaining structural integrity and enhancing resistance. It also analyses the protective mechanisms that fortify *S. aureus* biofilms against antimicrobial treatments. Furthermore, the review provides a comprehensive overview of recent therapeutic innovations, including enzymatic therapy, nanotechnology, gene editing, and phage therapy.

**Key scientific concepts of review:**

Emerging therapeutic strategies present novel approaches to combat *S. aureus* biofilm-associated infections through various mechanisms. This review discusses recent advancements in these therapies, their practical challenges in clinical application, and provides an in-depth analysis of each strategy’s mechanisms and therapeutic potential. By mapping future research directions, this review aims to refine anti-biofilm strategies to control infection progression and effectively mitigate recurrence.

## Highlights

• This review focuses on the structural characteristics, formation mechanisms, and antibiotic resistance of *S. aureus* biofilms.
• This review emphasizes overcoming the antibiotic resistance barrier of biofilms through multidimensional therapeutic approaches.
• This review highlights the synergy and integration of precision therapeutic strategies for biofilms.
• This review builds a complete theoretical structural system of *S. aureus* biofilms from micro-mechanisms to macro-therapeutics.

## Graphical Abstract

**Figure:**
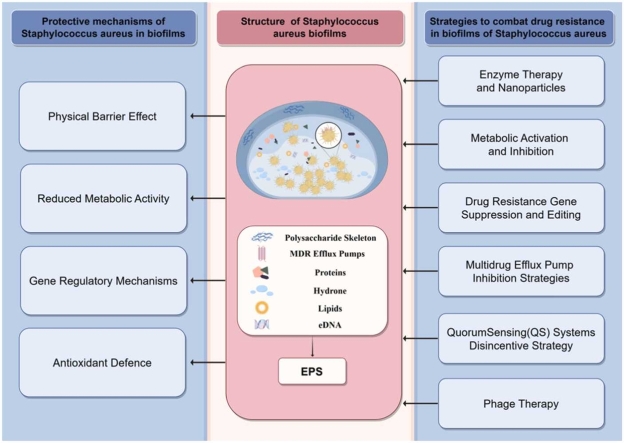

## 1 Introduction

*Staphylococcus aureus* (*S*. *aureus*) is a major pathogen responsible for a broad spectrum of infections worldwide, ranging from mild skin lesions to life-threatening systemic conditions [1]. In orthopedics, *S*. *aureus* is a leading cause of surgical site infections and implant-related infections [2], [3]. These persistent infections are often closely associated with biofilm formation, which not only promotes bacterial survival but also renders the infection highly resistant to conventional antibiotic treatments, resulting in chronicity and recurrence [4].

The treatment of chronic and refractory infections typically requires more than a single antibiotic regimen, necessitating additional interventions such as surgical debridement, implant removal, extended antibiotic courses, and, in some cases, re-implantation surgery [5]. Despite these combined efforts, achieving complete eradication of infections chronicized by biofilm formation remains a significant challenge, accompanied by high recurrence rates and substantial patient suffering [6]. The persistent and recurrent nature of biofilm-associated infections thus presents a profound challenge in clinical practice. These infections not only compromise patient outcomes but also impose a significant burden on healthcare systems. Frequent hospitalizations, complex surgeries, and prolonged antibiotic use increase healthcare costs and foster the emergence of antibiotic-resistant strains, complicating infection management and undermining antibiotic stewardship efforts [7]. Therefore, early detection, effective prevention of biofilm formation, and precise, efficient elimination of established biofilms are critical focal points for future clinical and research initiatives.

In recent years, researchers have explored a range of novel therapeutic strategies to address these challenges, including enzyme therapy, nanotechnology, gene editing, immunotherapy, and smart drug delivery systems. These innovative approaches aim to improve therapeutic efficacy by directly disrupting biofilm structures, enhancing antibiotic penetration, or targeting biofilm-specific genes [8], [9], [10], [11]. However, clinical infections often involve the synergistic interaction of multiple microorganisms. Most studies on polymicrobial interactions involving *S*. *aureus* are currently based on in vitro models that utilize fully mixed systems, which fail to systematically replicate the complex environment of clinical infections [12], posing significant challenges for the translation of these emerging therapeutic approaches into clinical applications.

This review provides a comprehensive overview of recent advances in *S*. *aureus* biofilms research, focusing on their structural characteristics, formation process, and mechanisms of resistance. It also discusses both established and emerging therapeutic strategies aimed at biofilm eradication. Special attention is given to innovative, multidimensional approaches designed to overcome the resistance barrier posed by biofilms. By emphasizing the synergistic and integrated nature of biofilm disruption strategies, this review offers a novel perspective on improving the management of biofilm-associated infections through precision medicine. It constructs a comprehensive theoretical framework, bridging microscopic mechanisms with macroscopic therapeutic strategies for *S*. *aureus* biofilms. The review aims to provide clinicians and researchers with a clear theoretical foundation, enhancing their understanding of the complexities of biofilm-related infections and inspiring future directions in both research and clinical practice.

## 2 Structure and formation of *S*. *aureus* biofilms

### 2.1 The basic components of biofilms and their physical properties

**Fig. 1:**
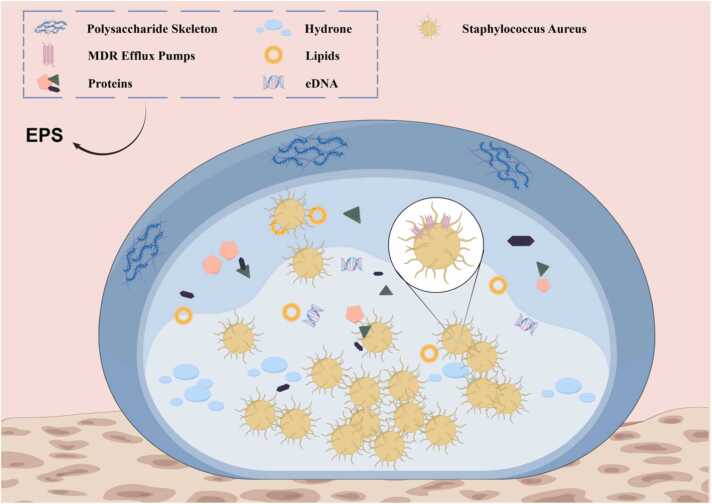
Compositional structure of biofilm in *Staphylococcus aureus*.

*S*. *aureus* biofilm is a complex microbial structure consisting of bacterial cells, extracellular polymeric substances (EPS), water, enzymes, and various biomolecules [13] (Fig. 1). The extracellular polymeric matrix, primarily composed of polysaccharides, proteins, lipids, and extracellular DNA (eDNA), is the core component of the biofilm [14]. The main EPS component in *S. aureus* biofilms is polysaccharide intercellular adhesin (PIA) [15], which plays a pivotal role in colonization, biofilm formation, immune evasion, antimicrobial resistance, and protection against phagocytosis [16]. The *ica* operon (*icaA*, *icaD*, *icaB*, and *icaC*) governs PIA synthesis, with factors such as anaerobic conditions, glucose, alcohol concentration, and antibiotics influencing its expression [17], [18], [19]. However, recent studies suggest that alternative biofilm forms independent of PIA exist. A small proportion of *S. aureus* strains can form biofilms even without the ica locus, and some strains with the locus continue biofilm production even after its deletion, indicating the presence of ica-independent pathways [15]. Polysaccharides and eDNA form the matrix's backbone, providing structural integrity and mechanical stability to the biofilm. eDNA production is closely linked to cell lysis. Studies show that the *cidA* and *Pfs* genes regulate autolysis in *S. aureus*, promoting cell lysis and the release of eDNA [20], [21]. In the early stages of biofilm formation, eDNA forms a mesh-like structure that supports bacterial adhesion. Without eDNA release, biofilm formation is significantly impaired. *S*. *aureus* biofilms also contain a variety of proteins, including surface protein A (Spa), fibrinogen-binding proteins A and B (FnBP A and B) [22], *S. aureus* surface protein G (SasG) [23], serine aspartate repeat protein (SdrC) [24], and clumping factor B (ClfB) [25]. These proteins have functions such as bacterial adhesion, signaling, and dynamic regulation [26].

The physical properties of biofilms greatly enhance the survival of *S*. *aureus*. The highly adhesive nature of biofilms enables *S. aureus* to strongly adhere to surfaces such as biomaterials, host tissues, and even in liquid environments [27]. Additionally, the polysaccharides and proteins within the biofilm matrix not only limit the diffusion of antibiotics but also serve as a primary immune evasion strategy [28]. This mechanism involves masking pathogen-associated molecular patterns (PAMPs) on the bacterial surface, preventing recognition by pattern-recognition receptors (PRRs) on host cells [29]. Furthermore, the altered physiology of biofilm cells reflects a unique microenvironment, where high cell density limits nutrient and oxygen availability, forcing cells into a quiescent metabolic state that enhances bacterial persistence. This dense bacterial arrangement and reduced metabolic activity make biofilms significantly more resistant to antibiotics [30].

The structural and physical properties of biofilms not only enhance the survival of *S*. *aureus*, but also enable it to establish persistent infection foci within the host. The complexity and stability of the biofilm structure contribute to the failure of conventional antibiotic therapies and present a significant challenge in treating biofilm-associated infections. As research advances on biofilm composition and functional properties, developing therapeutic strategies that disrupt or inhibit biofilm formation has become a key focus in anti-infective research.

### 2.2 The process of biofilm formation and its role in infection

**Fig. 2:**
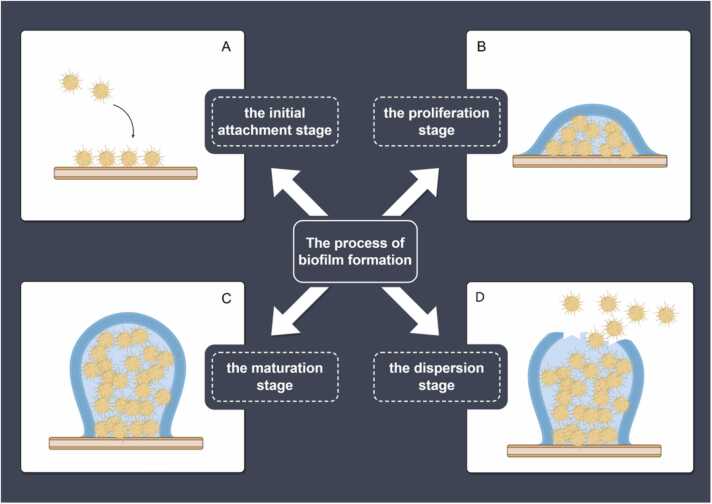
The process of Staphylococcus aureus biofilm formation.

**Fig. 3:**
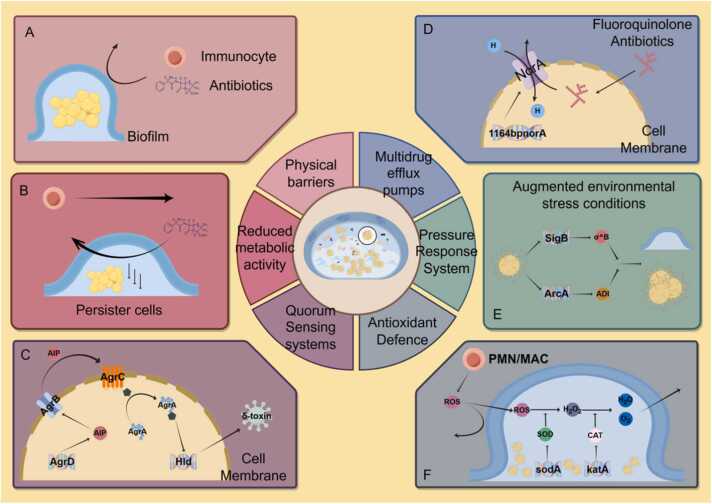
Protective mechanisms of biofilms in *Staphylococcus aureus*.

*S*. *aureus* biofilm formation is a complex and dynamic process. Its development is commonly categorized into three major phases: initial attachment, maturation, and dispersion [31]. Notably, Moormeier et al. identified two additional stages, termed “multiplication” and “exodus”, using the BioFlux1000 system [32]. However, the biological significance and mechanistic role of the exodus phase remain unclear. It seems to act as a trigger for biofilm maturation rather than as an independent stage. Therefore, we propose defining the developmental stages of *S. aureus* biofilms into four phases: (1) initial attachment (Fig. 2A), (2) proliferation, or irreversible attachment (Fig. 2B), (3) maturation (Fig. 2C), and (4) dispersion (Fig. 2D). Each phase is critical for bacterial survival, multiplication, and immune evasion.

The initial attachment phase is the first step in biofilm formation [33] and relies on compatible attraction forces. On biological surfaces, this attachment is typically mediated by protein-protein interactions. On abiotic surfaces, forces such as van der Waals interactions, electrostatic forces, and spatial effects facilitate bacterial adhesion [34]. When *S. aureus* contacts host tissues or implant surfaces, several components mediate the interaction. These include cell wall-anchoring (CWA) proteins, polysaccharides, agglutination factors ClfA and ClfB, and the negatively charged wall teichoic acid (WTA) [35]. These molecules mediate bacterial adhesion through intermolecular interactions and covalent attachment [34]. Notably, bacterial attachment during this phase remains reversible, allowing detachment and reversion to a free-living state under certain conditions. However, if the environment supports survival, *S. aureus* begins secreting EPS to enhance adhesion, transitioning into the irreversible attachment phase.

The proliferation phase is a critical stabilization stage, during which initial adhesion is reinforced. This reinforcement protects the cells from shear forces in flowing fluids, facilitating their transition into the irreversible attachment state. Consequently, this phase is also known as the irreversible attachment phase. A key feature of this stage is the initiation of gene expression changes mediated by quorum sensing (QS) signaling pathways. Bacteria secrete adhesive molecules (e.g., dextran), extracellular proteins, DNA, and other components to form the EPS, providing structural support, protection, and maintaining colony stability within the biofilm. This process firmly anchors the bacteria to the surface, leading to the formation of a microscopic colony, and making adhesion irreversible [27].

Biofilm maturation is a pivotal stage in biofilm formation. With continuous EPS secretion, the bacterial population becomes encapsulated, forming an organized and complex three-dimensional structure [32]. During this phase, the biofilm thickens, reaching its peak at maturation. Afterward, the metabolic activity of bacteria within the biofilm gradually decreases, leading to a relatively dormant state [30]. Moormeier and his team observed that the biofilm maturation phase also holds additional biological significance. The biofilm environment exhibits heterogeneity due to gradients in oxygen, nutrients, and metabolic waste, creating distinct microniches. This heterogeneity drives metabolic differentiation among bacteria in different microniches and promotes cooperation between subpopulations. For example, outer-layer cells undergo lysis and release eDNA to support structural stability, while inner-layer cells utilize these resources to adapt to low-energy environments, further contributing to persister cell formation. Moreover, spatial and physiological heterogeneity creates protective barriers that limit the penetration and efficacy of antimicrobial agents, significantly enhancing the biofilm's resistance to antibiotics [32].

The dispersion phase is the final step in the biofilm life cycle. Under certain conditions, such as nutrient depletion, environmental stresses, or altered signals perceived by the bacterial community, a portion of the bacteria detaches from the substrate and reverts to a planktonic state. This allows them to spread to new sites, perpetuating the infection and ensuring survival in more favorable environments [36]. This stage is critical for the dissemination of *S*. *aureus* and the progression of infection. It enables the bacteria to establish multiple foci of infection within the host, leading to recurrence or further spread [31].

## 3 Protective mechanisms of *S*. *aureus* in biofilms

### 3.1 Physical barrier effect

The physical barrier of *S*. *aureus* biofilm serves as a primary defense mechanism against external threats, primarily relying on the EPS secreted by the bacteria. This matrix provides structural support and plays a crucial role in protecting against antibiotics and immune cells, enabling *S*. *aureus* to persist within the host [28], [34], [37].

The biofilm matrix creates a robust barrier through an intricate network of polysaccharides, proteins, and lipids. Its primary function is to physically impede antibiotic penetration [38]. Due to the dense, viscous nature of the biofilm matrix, antibiotics struggle to effectively penetrate and reach the bacteria at their site of residence (Fig. 3A). However, this effect may vary depending on the type of antibiotic [39]. Furthermore, the extracellular matrix (ECM) serves as a reservoir for antibiotic-inactivating enzymes (e.g. BlaZ, aphA, aadD, and aacA-aphD). Even if small amounts of antibiotics manage to cross the biofilm, they may be sequestered or neutralized within the matrix, reducing the effective concentration further [40]. This barrier effect prevents antibiotics from achieving bactericidal or bacteriostatic effects, prolonging bacterial exposure to sub-inhibitory concentrations and fostering conditions that promote antibiotic resistance. Biofilms also restrict the diffusion of nutrients and metabolic waste, creating a low-oxygen, low-pH microenvironment within the biofilm [41]. This microenvironment impairs antibiotic efficacy, as the activity of certain antibiotics is significantly reduced under low-oxygen conditions, and low pH can compromise the molecular stability and permeability of antibiotics. In addition, since *S. aureus* is often co-isolated with other bacteria or fungi in clinical infections, the role of polymicrobial biofilms cannot be overlooked. Studies have shown that when *Pseudomonas aeruginosa* coexists with *S. aureus*, the biomass of the mixed biofilm increases significantly [42]. Furthermore, research by Kong et al. revealed that the hyphal structure of *Candida albicans* provides a physical scaffold for *S. aureus*, promoting surface attachment and significantly enhancing biofilm formation. Compared with mono-species *S. aureus* biofilms, mixed biofilms exhibit substantially increased biomass and structural complexity [43]. This mixed biofilm structure further strengthens the physical barrier effect, posing additional challenges to antimicrobial treatments.

In addition to obstructing antibiotic penetration, the physical barrier of the biofilm effectively hinders the ability of host immune cells, including neutrophils and macrophages, to penetrate the matrix and reach *S*. *aureus*. Hamid et al. demonstrated that *S*. *aureus* biofilms can slow neutrophil migration, impair phagocytic activity, and reduce the production of reactive oxygen species (ROS), significantly diminishing their bactericidal capacity [44]. A study by Benjamin P. Howden et al. found that *S*. *aureus* biofilms skewed the host immune response towards an anti-inflammatory state, promoting bacterial persistence rather than clearance [28]. Scherr et al. showed that biofilms can downregulate the expression of key genes involved in metabolism and cell wall synthesis, impairing the bactericidal function of macrophages [45]. These findings suggest that biofilms modulate immune cell function through gene regulation, further reinforcing the mechanisms by which biofilm barriers facilitate immune evasion.

### 3.2 Reduced metabolic activity

The reduced metabolic activity of *S*. *aureus* within biofilms is a key mechanism contributing to its resistance. During biofilm formation, the bacteria gradually enter a hypometabolic state, which supports long-term survival and significantly enhances their resistance to antibiotics [46]. This hypometabolic state allows the bacteria to evade drug action through various pathways, contributing to the persistence and refractory nature of biofilm-associated infections (Fig. 3B).

The bactericidal effect of antibiotics primarily relies on active bacterial metabolism. Many commonly used antibiotics, such as beta-lactams and aminoglycosides, exert their effects by interfering with bacterial cell wall or protein synthesis—processes that are significantly slowed or halted in a hypometabolic state. Consequently, when *S*. *aureus* enters this state, the antibiotic's target becomes less active, significantly reducing its bactericidal efficacy [47].

Bacteria in a hypometabolic state exhibit increased tolerance to antibiotic-induced oxidative stress. In metabolically active bacteria, the action of antibiotics often generates significant amounts of ROS, which can damage bacterial DNA, proteins, and membrane structures, ultimately leading to cell death [48]. However, bacteria in a hypometabolic state are less susceptible to oxidative damage, partly because their reduced metabolic activity results in fewer endogenous ROS. Additionally, antioxidant enzymes within the biofilm neutralize ROS accumulation, further protecting the bacteria from lethal damage [49].

The hypometabolic state is also closely linked to bacterial persistence [50]. Persister cells represent a small subpopulation of bacteria within the biofilm that exist in a very low metabolic or dormant state, exhibiting high levels of antibiotic resistance. These cells survive antibiotic treatment and, although they do not carry traditional resistance genes, their metabolic state allows them to evade antibiotic-mediated killing [51]. Once antibiotic treatment is discontinued, persister cells can reactivate and proliferate, leading to the recurrence of the infection.

### 3.3 Gene regulatory mechanisms

The formation and maintenance of *S*. *aureus* biofilms depend on a complex network of gene regulatory mechanisms that govern not only the biofilm's structure and function, but also confer high drug resistance and adaptability to the bacteria. Changes in the expression of specific genes during biofilm formation influence bacterial survival strategies at multiple levels, enabling them to evade host immune defenses and withstand antibiotic treatments.

#### 3.3.1 QS systems

QS is one of the central mechanisms by which *S*. *aureus* regulates biofilm formation and maintenance. Through the QS system, bacteria detect cell density in their local environment. Once this density reaches a critical threshold, the concentration of signaling molecules (e. g., autoinducing peptide, AIP) increases, activating the QS system and triggering a cascade of gene expression changes [52], [53]. This regulatory mechanism plays a crucial role in biofilm formation, maturation, and stable maintenance.

In *S*. *aureus*, the central component of the QS system is the agr system (accessory gene regulator). The agr operon is organized around two divergent promoters, P2 and P3, generating two primary transcripts, *RNAII* and *RNAIII*, respectively [54], [55]. *RNAII* encodes *AgrB*, *AgrD*, *AgrC*, and *AgrA*. The agr system regulates the expression of various biofilm-related genes by detecting and modulating the concentration of signaling molecules, specifically AIP. AIP is encoded by *AgrD* (as a propeptide) and released into the extracellular space via processing by *AgrB* (an integral membrane protein) [56]. During biofilm maturation, AIP accumulates in the extracellular environment, eventually reaching a threshold concentration that activates the two-component system comprising *AgrC* and *AgrA* through phosphorylation. This activation in turn triggers *RNAIII* transcription from the P3 promoter [57]. *RNAIII* mediates the upregulation of virulence factor secretion and encodes the δ-toxin (*hld*) gene (Fig. 3C), a potent surfactant that prevents microbial adhesion to surfaces [58]. This suggests that activation of the agr system may facilitate biofilm dispersion, allowing bacteria to detach from the biofilm and spread to new infection sites [59]. Under specific conditions, such as nutrient depletion or increased environmental stress, the QS system triggers biofilm dispersion, releasing bacteria into a planktonic state. This process allows the bacteria to spread to new host sites or escape unfavorable environments, sustaining the persistence of the infection.

The QS system not only regulates biofilm formation but is also essential for its maintenance. For instance, when biofilms are exposed to antibiotics or the immune system, the QS system modulates the expression of drug resistance genes by upregulating multidrug efflux pumps or activating antioxidant defenses. These responses help biofilms preserve their integrity and functionality under external stress, thereby enhancing bacterial survival [53]. The roles of multidrug efflux pumps and antioxidant defenses in biofilm resilience will be further discussed in subsequent sections.

The QS system of *S. aureus* is regulated not only by environmental factors and immune components but also by physicochemical conditions. A recent study demonstrated that the QS system follows a heterogeneous activation pattern under fluidic conditions [60]. Under high-flow conditions, QS activity is largely suppressed. In regions with little or no fluid flow, AIP signaling accumulates, leading to the subsequent activation of the QS system. Additionally, high magnesium concentrations have been shown to downregulate the QS system, potentially promoting *S. aureus* biofilm formation [61]. These findings highlight that, although the relationship between the QS system and biofilm formation has been extensively studied, many aspects remain unexplored. For instance, it is still unclear how biofilm formation is influenced by the QS system or other regulatory systems across different infection sites, and which environmental cues drive *S. aureus* biofilm-associated infections.

#### 3.3.2 Multidrug efflux pumps and drug resistance systems

The multidrug efflux pump is a key resistance mechanism in *S*. *aureus* biofilms, comprising several systems, including *QacA*, *NorA*, *TetA(K)*, *Tet38*, *MdeA*, and *SdrM* [62]. Among these, *NorA* is a core gene in *S*. *aureus*, encoded by the *1164 bp NorA* gene [63]. Within biofilms, *S*. *aureus* significantly upregulates the expression and activity of efflux pump genes through the QS system or other regulatory mechanisms [64]. Efflux pumps contribute to biofilm formation by exporting quorum-sensing molecules necessary for biofilm development. They also enhance tolerance to antimicrobial compounds and modulate gene expression involved in ECM production [65].

These efflux pumps utilize proton motive force to actively export antibiotic molecules from the bacterial cell, reducing intracellular antibiotic accumulation and significantly diminishing the drug's bactericidal effect [66]. For example, norfloxacin and other fluoroquinolone antibiotics are typical substrates of the *NorA* efflux pump [67], [68]. These antibiotics typically kill bacteria by interfering with DNA synthesis, but when *NorA* expels the antibiotic rapidly, the intracellular concentration drops dramatically. This prevents the drug from reaching a sufficient bactericidal concentration, enabling bacterial survival [67](Fig. 3D).

Additionally, *S*. *aureus* enhances its survival in biofilms by regulating drug resistance-associated genes. The enzymes encoded by these genes can hydrolyze antibiotics to inactivate them or chemically modify them to render them ineffective [69]. This mechanism leads to resistance against multiple antimicrobial drugs. For example, within the biofilm environment, significantly higher expression levels of the *mecA* gene confer resistance to β-lactam antibiotics in *S*. *aureus*, indicating increased resistance to β-lactams in *S*. *aureus* biofilms [70], [71]. Similarly, the *mecC* gene, a homolog of *mecA*, encodes PBP2a, which mediates β-lactam resistance in *S. aureus* [72].

#### 3.3.3 Pressure response system

Another key regulatory mechanism involves environmental stress response systems. These are genes activated in response to adverse conditions such as oxidative stress, nutrient depletion, and pH changes. In biofilms, *S*. *aureus* is often exposed to harsh environments, including low oxygen levels and low pH. Genes like *sigB* enable *S*. *aureus* to adapt to these conditions by regulating the expression of stress response proteins, thereby maintaining biofilm integrity and functionality. Additionally, the study by Resch et al. showed that the arc gene cluster is expressed at higher levels in biofilm cells after 16, 24, and 48 hours of growth. These genes are essential for late-stage cellular metabolism. As shown in Fig. 3E, when certain biofilm regions become hypoxic, the arginine deiminase (ADI) encoded by this gene enables cells to obtain ATP by converting arginine into citrulline [73], allowing survival and biofilm stability under resource-limited conditions.

### 3.4 Antioxidant defence

The antioxidant defense mechanisms in *S*. *aureus* biofilms are crucial for resisting host immune attacks. During infection, the host immune response kills pathogenic bacteria by producing ROS through neutrophils and macrophages [74]. These ROS, including hydrogen peroxide, superoxide, and hydroxyl radicals, damage bacterial DNA, proteins, and lipid membranes via oxidative stress. However, *S*. *aureus* survives immune attacks by activating various antioxidant defense mechanisms within its biofilm, enhancing its resistance to ROS.

Bacteria within biofilms upregulate the expression of antioxidant enzymes, including superoxide dismutase (SOD) and catalase (CAT). SOD rapidly converts superoxide into less reactive hydrogen peroxide, which is then broken down by CAT into water and oxygen [49] (Fig. 3F). The synergistic action of these enzymes significantly reduces ROS accumulation within the biofilm, mitigating oxidative stress damage. Notably, upregulated bacterial pigments also protect in polymicrobial biofilms. Studies show that when *Pseudomonas aeruginosa* coexists with *S. aureus*, it enhances the production of the pigmented virulence factor staphyloxanthin (STX), a potent antioxidant that further strengthens *S. aureus* resistance to oxidative stress [75]. Acting as a potent antioxidant, STX further strengthens the resistance of *S. aureus* to oxidative stress through its remarkable antioxidative properties, thereby providing an additional survival advantage in complex microbial environments.

Additionally, the EPS of the biofilm itself acts as an antioxidant. Polysaccharides and proteins in the EPS not only serve as a physical barrier against ROS but also reduce their activity by binding to or neutralizing them [29]. The multilayered structure of the biofilm helps buffer and diffuse ROS attacks. Bacteria in the outer layers absorb most oxidative stress from ROS, protecting bacteria in the inner layers [44].

ROS not only cause cellular damage but also induce genetic changes, potentially leading to drug-resistant mutations. Ryder et al. demonstrated that mutation frequency in *S. aureus* significantly increases in the biofilm state, up to 60-fold higher than in the planktonic state. This heightened mutability is linked to antibiotic resistance, especially during the early stages of biofilm formation, accelerating the development of resistance mutations [76].

The regulation of antioxidant genes in biofilms plays a crucial role in bacterial survival. For example, the *sigB* gene in *S*. *aureus* activates the expression of antioxidant-related genes, such as *katA* (catalase) and *asp23*, in response to oxidative stress, enhancing bacterial survival under such conditions [77], [78]. The upregulation of genes like *sigB* further strengthens the bacterial antioxidant defense system in the biofilm, enabling it to withstand intense immune pressure [79].

## 4 Strategies to combat drug resistance in biofilms of *S*. *aureus*

As antibiotic resistance in *S*. *aureus* biofilm-associated infections intensifies, researchers are exploring various innovative strategies to address this clinical challenge. These strategies aim to disrupt biofilm structure and enhance antibiotic efficacy. This section outlines several effective approaches, including enzyme therapy combined with nanoparticle technology, metabolic activation, resistance gene suppression, multidrug efflux pump inhibition, QS inhibition, and phage therapy (Table 1). These methods provide new insights and potential solutions for treating *S*. *aureus* infections.

**Table 1:** Strategies against intra-biofilm *Staphylococcus aureus* resistance and their mechanisms of action.

Strategy category	Substance that acts		Mechanism of action	Reference
**Enzyme therapy**	Glycoside hydrolases		Destruction of polysaccharide components in EPS, weakening the barrier function of biofilm	[80], [81]
	DNase		Degradation of eDNA in *S*. *aureus* biofilms, disrupting the physical structure of the biofilm	[82]
	Protein hydrolysing enzymes		Degradation of structural and functional proteins in biofilms, causing further loss of protective capacity of the biofilm	[83], [84], [85]
**Nanotechnology**	Drug-carrying nanoparticles		Enhancing drug permeability in biofilms by loading different drugs provides sustained release and synergises antimicrobial activity in many ways depending on the type of drug	[88], [89]、[94], [95], [96]
	Ag, Cu, Au and other metal nanoparticles		Killing of bacteria through various mechanisms, such as ROS production, disruption of bacterial cell membranes, inhibition of bacterial metabolic activities, etc.	[90], [91], [92], [93]
	Smart nanoparticles		Responding to specific conditions in the biofilm microenvironment (e. g. acidic pH, temperature, or redox state) to enable targeted release of antibiotics	[97], [98]
**Metabolic activation**	uracil		Facilitates antibiotic uptake by enhancing TCA and producing more NADH and PMF Enhances bacterial respiratory activity and increases ROS production	[102]
	JBD1		Activates the respiratory activity of *S*. *aureus* and increases its susceptibility to antibiotics	[103]
**Metabolic repression**	Cu-POM		Formation of hydroxyl radicals that trigger bacterial copper overload and inhibit the tricarboxylic acid cycle (TCA) in *S*. *aureus*, leading to the accumulation of peroxides, which in turn trigger bacterial death similar to copper poisoning	[104]
	MnSe2		Releases volatile hydrogen selenide (H2Se), which disrupts bacterial metabolic homeostasis, inhibits protein synthesis, and interferes with basic bacterial metabolic pathways, leading to bacterial death	[105]
	Nb2C@TP		Activation of co-regulatory genes to prevent bacterial attachment and promote shedding of formed biofilms; down-regulation of bacterial energy metabolism pathways, such as TCA and phosphotransferase system (PTS), to directly kill MRSA	[106]
**Gene editing technology**	CRISPR-*cas9*		Deletion or modification of drug-resistant genes by specific recognition and cleavage of target DNA sequences, thereby directly disrupting their function	[108], [109], [111]
**Multidrug efflux pump suppression**	Indoles, omeprazole, piperine analogues, plant extracts		Inhibition of *S*. *aureus* efflux pump activity increases the cumulative concentration of antibiotics in bacteria and weakens resistance	[112], [113], [114], [115], [116], [117], [118]
**QS systems disincentive**	Natural plant extracts	pyranoanthocyanin	Inhibition of the Agr system in *S*. *aureus* significantly down-regulates *AgrA* gene expression	[120], [121]
		carnosic acid and carnosol	Inhibition of *AgrC* binding to AIP interfered with the activation process of *AgrA*, reducing the binding of *AgrA* to promoters P2 and P3, which in turn inhibited the expression of *RNAIII* and *psmα* genes	[122]
		Physalin H、 B and isophysalin B	Inhibition of *AgrA* function reduces *AgrA* binding to promoters P2 and P3, which in turn inhibits *RNAIII* and *psmα* gene expression	[123]
	AIP analogue		Competitively binds to *AgrC*, preventing the binding of natural AIP signalling, which in turn inhibits QS signalling and reduces biofilm formation and release of virulence factors	[124], [125]
**Phage therapy**	Phages and their degrading enzymes		Phage lyses bacteria and disrupts the biofilm, carrying degrading enzymes that further destabilise the biofilm	[129], [130], [131], [132], [133]

### 4.1 Enzyme therapy and nanoparticles

Enzyme therapy has emerged as an innovative strategy against *S*. *aureus* biofilms. It aims to directly disrupt the biofilm structure through specific enzymes, weakening its defenses and making the bacteria more susceptible to antibiotics or the host immune system.

The polysaccharide components of *S*. *aureus* biofilms play crucial roles in biofilm formation and integrity. They act as a protective barrier against antimicrobial agents and environmental stressors [14]. As a result, the active degradation of these polysaccharide components has become a potentially broad-spectrum approach to combating *S*. *aureus* biofilm infections. A study by Derek Fleming et al. found that glycoside hydrolases, particularly α-amylase and cellulase, were effective in degrading biofilms formed by *S*. *aureus,* promoting the dispersion of bacteria into a planktonic state, which made them more susceptible to antibiotics [80]. In addition, Redman et al. (2021) reported that enzymes such as alginate lyase, pectinase, amyloglucosidase, inulinase, and xylanase also significantly disrupted *S. aureus* biofilms. Their study further demonstrated that these enzymes could enhance the efficacy of antibiotics, such as meropenem, providing a complementary strategy for treating biofilm-associated infections [81]. However, studies have shown that these glycoside hydrolases (GHs) are less effective in disrupting multi-species biofilms. This suggests that, when dealing with complex multi-species biofilms in clinical settings, enzyme mixtures targeting different types of polysaccharide linkages may be key for future research.

Additionally, DNase (deoxyribonuclease) is commonly used because eDNA functions as a structural scaffold within the EPS, and its degradation disrupts the biofilm's physical structure, weakening its protective function [8]. Lacey et al. (2023) found that the synergistic effect of DNase I and DNase I L3 reduced the severity of *S*. *aureus* infection and significantly decreased biofilm formation [82]. Protein hydrolases, on the other hand, compromise the biofilm's protective capacity by degrading structural and functional proteins within the biofilm [83], [84], [85].

Notably, the combined use of these enzymes can produce a synergistic effect, significantly enhancing biofilm destruction by targeting multiple pathways. For example, a study by Devlin et al. demonstrated that a combination of three enzymes—lysostaphin, serrapeptase, and DNase I—significantly increased biofilm removal efficiency, particularly in MRSA biofilms, achieving near-complete biofilm removal and outperforming the use of individual enzymes [86]. However, this study did not address multi-species biofilms. Whether the combined use of different types of enzymes is more effective in multi-species biofilms remains to be explored further.

The application of nanoparticle technology in biofilm therapy has demonstrated significant potential and has become a key area of research in recent years. Nanotechnology offers several advantages over conventional treatments. For instance, materials with larger surface area-to-volume ratios exhibit higher bioreactivity and are less susceptible to enzymatic degradation, drug toxicity, or untargeted delivery. This makes them ideal carriers for enzymes and antibiotics [87]. The combination of nanoparticles with enzymes or antibiotics provides a dual approach to combating biofilms. It effectively disrupts the biofilm’s physical barrier while allowing antibiotics or other antimicrobial agents to penetrate deeper into the biofilm, thereby effectively killing the bacteria. For example, Weldrick et al. demonstrated that functionalized nanocarriers carrying proteases and ciprofloxacin significantly enhanced *S*. *aureus* biofilm removal, not only degrading the protein matrix of the biofilm but also reducing biofilm residues by over 50 % [88]. A 2023 study by Li et al. developed a DNase I and vancomycin hydrogel nano-delivery vehicle that effectively eliminated MRSA infections, prevented biofilm formation, and provided a sterile environment to promote healing in osteoporotic fracture-associated infections [89].

Additionally, metallic nanoparticles (e. g., silver, gold, and copper) can serve as carriers for antibiotics and exhibit independent antibacterial activity. For instance, Katharina Richtr et al. reported that colloidal quasi-spherical AgNPs significantly eradicated biofilms in MRSA isolates (97 % ± 1 %) [90]. In another study, multivalent aminoglycoside-based gold nanoparticles (AuNPs) were synthesized by modifying AuNPs with D-glucosamine (GluN). These gold nanoparticles displayed significant efficacy in targeting biofilm-embedded bacteria, particularly in the treatment of MRSA-infected skin wounds, while also showing high biocompatibility [91]. However, copper ions require elevated concentrations to achieve optimal bactericidal effects, which can result in toxicity to mammalian cells. To address this, Kannan et al. developed multilamellar liposomes encapsulating lipopeptides and copper nanoparticles (CuNPs). This novel formulation demonstrated significant antimicrobial activity against MRSA in both planktonic and biofilm states. The liposomal encapsulation not only enhanced the pharmacokinetics and pharmacodynamics of CuNPs but also minimized their potential toxicity. The synergistic interaction between CuNPs and lipopeptides significantly reduced EPS production (by 47 %) and markedly increased intracellular ROS generation (by 75 %). These actions compromised biofilm stability, thus improving bacterial eradication [92]. Metallic nanoparticles can kill bacteria through various mechanisms, including ROS generation, bacterial membrane disruption, and inhibition of bacterial metabolic activity [93]. Additionally, when combined with antibiotics, nanoparticles often produce a synergistic effect, greatly enhancing therapeutic efficacy. For instance, silver nanoparticles were significantly more effective against *S*. *aureus* biofilms when used with vancomycin compared to antibiotics alone.

In addition, combining nanoparticles with quorum quenching (QQ) technology allows for targeting and interference with the QS system in *S*. *aureus*, thereby facilitating antibacterial treatment. Quorum quenching agents, including QQ enzymes and QS inhibitors (QSIs), function by cleaving or competitively inhibiting signaling molecules within the QS system [94]. For example, savirin, a small-molecule inhibitor of *S*. *aureus* virulence, prevents the binding of *AgrA* protein to its promoter site, thereby blocking the stimulation of P2 and reducing the expression of regulated genes [95]. Nanoparticles derived from natural extracts, such as phenylbutyric acid and baicalein, can specifically target *AgrB* and *AgrC*, respectively, inhibiting *RNAIII*-activating peptides and consequently preventing the phosphorylation of downstream cascades. This suppresses the expression of genes responsible for biofilm formation [96].

The development of smart nanoparticles (intelligent drug delivery systems) has further expanded possibilities for antibiotic therapy. These nanoparticles can respond to specific conditions in the biofilm microenvironment (such as acidic pH, temperature, or redox state), thus enabling the targeted release of antibiotics. For example, a pH-sensitive oxidized dextran-based dual-carrier hydrogel, which detects the acidic environment of the biofilm, undergoes structural changes that trigger the release of encapsulated sulphadiazine (SD) and tobramycin (TOB), directly targeting the bacteria and demonstrating high antibacterial activity, as evidenced by Zhang et al. [97]. A temperature-sensitive, dual-drug-loaded smart delivery system developed by Akhlaghi et al. undergoes rapid gelation at 37°C, enhancing the stability of the antimicrobial material in vivo. This allows the antibiotics to maintain long-term stability, achieving a biofilm clearance rate of up to 95. 88 % [98]. This targeted release not only increases the local concentration of the drug but also reduces systemic exposure and associated side effects. However, the preparation of these intelligent systems often involves complex chemical modifications or functionalization processes, which can result in instability, potentially limiting their reliability in clinical applications.

### 4.2 Metabolic activation and inhibition

Numerous studies have suggested that the metabolic state of bacteria plays a crucial role in mediating antibiotic resistance [99], [100]. Metabolic activation therapy seeks to target persister cells in biofilms, which exist in a low metabolic state, by modulating key metabolites to enhance their susceptibility to antibiotics [101]. By stimulating or enhancing specific metabolic pathways in *S*. *aureus*, bacteria in a dormant state can be reactivated, making them more responsive to antibiotics. For instance, a 2023 study by Fan et al. demonstrated that uracil facilitated metabolic reprogramming in *S*. *aureus*, enhancing the tricarboxylic acid (TCA) cycle, which resulted in increased NADH production and proton motive force (PMF), thereby promoting antibiotic uptake. Additionally, uracil boosted bacterial respiratory activity and significantly elevated ATP levels. This heightened respiratory activity was also accompanied by an increase in ROS, which is closely linked to bacterial cell death [102]. Similarly, Okuda et al. found that JBD1, a small molecule, enhanced *S. aureus*’s sensitivity to antibiotics, particularly aminoglycosides such as gentamicin, kanamycin, and tobramycin, by activating its respiratory processes [103]. These metabolic activators prompt the resumption of metabolic activity in persister cells, enabling otherwise drug-resistant bacteria to be efficiently eradicated during antibiotic treatment.

One of the primary advantages of the metabolic activation strategy is its ability not only to enhance antibiotic effectiveness but also to reduce persister cell formation, thereby lowering the risk of recurrent infections. By activating the metabolic state of *S. aureus*, biofilm integrity is compromised, and biofilm formation is further diminished through increased bacterial killing, demonstrating substantial clinical potential. Combining various therapeutic strategies can effectively counteract the multifaceted defense mechanisms of biofilms, while metabolic activation serves to amplify the bactericidal effects of antibiotics. Future clinical investigations may focus on optimizing these combination therapies and identifying more effective drug regimens and activators for different types of biofilm-associated infections.

In contrast, energy metabolism inhibitors can disrupt bacterial energy production, leading to energy depletion and cell death. A recent study revealed that copper-doped polyoxometalate nanoclusters (Cu-POM) could self-assemble in the acidic microenvironment of biofilms and generate toxic hydroxyl radicals through endogenous hydrogen peroxide (H_2_O_2_). This process boosted bacterial metabolic activity and enhanced the function of copper transport proteins, resulting in bacterial copper overload. The copper accumulation disrupted the TCA cycle in *S*. *aureus*, causing peroxide buildup, which led to bacterial death via copper toxicity and the effective disintegration of *S*. *aureus* biofilms [104]. Manganese diselenide (MnSe_2_) nanoparticles,as explored by He et al., release volatile hydrogen selenide (H_2_Se) in the acidic conditions of biofilms, disrupting bacterial metabolic homeostasis by integrating with the sulfur metabolic pathway. This disruption inhibits protein synthesis and affects key metabolic processes, ultimately resulting in bacterial death [105]. Additionally, photothermal therapy (PTT) enhances the antimicrobial effect of these treatments by raising the temperature. The inhibition of bacterial energy signaling remains an essential strategy in eliminating biofilms of drug-resistant bacteria and combating persistent infections. Yang et al. developed a biotherapeutic platform (Nb_2_C@TP) using two-dimensional niobium carbide nanosheets that activate co-regulatory genes (*AgrA*, *AgrB*, *sak*, *HtrA*) to prevent bacterial attachment and promote biofilm shedding, while concurrently downregulating bacterial energy metabolism pathways, such as the TCA cycle and the phosphotransferase system (PTS), thereby directly killing MRSA [106].

### 4.3 Drug resistance gene suppression and editing

As drug resistance increases in *S*. *aureus* biofilm-associated infections, gene suppression and editing techniques targeting drug-resistant genes have become critical in combating these persistent infections. The high expression of resistance genes, such as *mecA* and multidrug efflux pump genes (e. g., *NorA*), within biofilms significantly contributes to the failure of antibiotic therapy [107]. Therefore, inhibiting the expression or function of these genes has become a primary focus of therapeutic strategies.

Gene suppression strategies mainly involve interfering with regulatory pathways or directly inhibiting gene expression. Among these, gene editing technologies, particularly the CRISPR-*cas9* system, have shown considerable promise in targeting drug-resistant genes. CRISPR-*cas9* can precisely delete or modify drug-resistant genes by recognizing and cleaving specific DNA sequences, thereby disrupting their function [10]. For instance, Kang et al. successfully targeted and cleaved the *mecA* gene in *S*. *aureus* using nanocomplexes as CRISPR-*cas9* vectors, resensitizing the strain to β-lactam antibiotics [108]. Additionally, Bikard et al. employed phage delivery of the *cas9* gene and its RNA guide sequences to target the *mecA* gene in MRSA [109], demonstrating therapeutic efficacy in a mouse skin colonization model. Although these studies highlight the significant potential of the CRISPR-*Cas9* system in targeting antibiotic resistance genes in S. aureus, their effects on bacterial biofilms remain underexplored.

Biofilm formation involves multiple genes, and targeting a single resistance gene may not significantly impact biofilm structure. Additionally, the bactericidal effect of CRISPR-*Cas9* relies heavily on its effective delivery to the target bacteria [110], and current delivery systems may hinder further research progress. Therefore, future studies should focus on developing more advanced CRISPR-*Cas9* systems and efficient delivery strategies. However, a study by Cobb et al. demonstrated that CRISPR-*Cas9* phages, at a concentration of 1 × 10^8^ pfu/mL, were effective in eliminating *S. aureus* biofilms in vitro [111]. This effect may be attributed to the use of a more efficient delivery system, such as alginate hydrogel, although the exact molecular mechanism remains unclear. It is possible that the biofilm inhibition observed was an indirect result of reduced bacterial adaptability following the destruction of resistance genes, rather than direct targeting of biofilm-related genes by CRISPR-*Cas9*. Future research may therefore focus on targeting genes involved in biofilm formation to more effectively modulate biofilm development and stability.

While gene suppression and editing techniques have shown great potential in laboratory studies, they still face challenges in clinical applications, such as efficient delivery to target cells and minimizing off-target effects [10]. However, as technology advances, these strategies hold promise for offering novel solutions in the treatment of biofilm-associated infections.

### 4.4 Multidrug efflux pump inhibition strategies

The multidrug efflux pump is a key mechanism of antibiotic resistance in *S*. *aureus* biofilms. It actively pumps antibiotics out of the bacterial cell, reducing the intracellular concentration of the antibiotic and thereby diminishing its bactericidal effect [66]. Additionally, efflux pumps are closely linked to bacterial toxicity and biofilm formation. One proposed molecular mechanism is that efflux pumps expel toxic compounds, maintaining bacterial viability and promoting biofilm formation [67]. Consequently, efflux pump inhibitors (EPIs) have the potential to restore antibiotic activity, making them a promising approach to overcoming bacterial resistance.

Various compounds capable of inhibiting efflux pump activity have been identified. These inhibitors act through different mechanisms. For instance, conventional inhibitors like omeprazole and indoles effectively inhibit the *NorA* efflux pump by disrupting the proton motive force, thus reducing bacterial drug resistance [112], [113]. More recently, synthetic and modified inhibitors have shown greater efficacy against drug-resistant pathogens. Kalia et al. synthesized piperine analogues (capsaicin), studied their quantitative structure-activity relationships, and evaluated their efflux pump inhibitory activity, finding them more effective than other EPIs, such as risperidone and verapamil [114]. N,N′-disubstituted cinnamic amides, synthesized by Radix et al., were also effective against *NorA*-overexpressing *S*. *aureus*, enhancing ciprofloxacin’s effect [115]. Additionally, plant extracts, including two flavonols (chrysosplenol-D and chrysoplenetin) from *Artemisia annua L*. [116], isoflavones from *Lupinus argenteus* [117], and chalcone and smoketree extracts (spinosan A and isoflavone) [118], exhibited significant inhibitory effects on *NorA* overexpression in *S. aureus*, enhancing antibiotic efficacy. Due to their lower toxicity and excellent biocompatibility, these natural-origin EPIs hold promising potential for antimicrobial therapy.

However, the specific role of the NorA efflux pump in biofilm formation is not fully understood. While some studies suggest that efflux pump inhibitors may interfere with biofilm formation, the underlying molecular mechanisms remain unclear. Therefore, future research is needed to explore these mechanisms and evaluate the clinical potential of efflux pump inhibitors.

### 4.5 Quorum sensing (QS) systems disincentive strategy

QS systems, pivotal for coordinating bacterial behaviors through density-dependent signaling, are crucial in the formation and maintenance of *S*. *aureus* biofilms and the expression of virulence factors [52], [119]. Inhibition of QS can thus reduce virulence factor production and biofilm formation, enhancing pathogen susceptibility to antimicrobial agents and aiding the host immune system in managing infections [11]. QS inhibitors (QSIs) disrupt biofilm development and expansion by interfering with the synthesis, release, or detection of bacterial signaling molecules, effectively curtailing inter-bacterial communication.

Numerous natural compounds have been identified as potent QS inhibitors. Correia et al. highlighted that pyranoanthocyanin, particularly carboxypyranocyanidin-3-O-glucoside, significantly inhibited *S*. *aureus* biofilm formation by targeting the Agr system and down-regulating *AgrA gene* expression. These compounds also suppressed other biofilm-related genes, such as ica, thereby mitigating biofilm production and drug resistance [120], [121]. Extracts from Rosmarinus officinalis L., particularly carnosic acid and carnosol, have been shown to inhibit *AgrA* activation by preventing *AgrC* from binding to AIP, reducing *AgrA* binding to promoters P2 and P3, and subsequently diminishing *RNAIII* and *psmα* gene expression, which curbs biofilm formation and bacterial virulence [122]. Similarly, a 2024 study by Chiba University found that Physalin H, Physalin B, and isophysalin B reduced *RNAIII* and *psmα* gene expression by inhibiting *AgrA* function [123]. Notably, these inhibitory compounds were predominantly derived from herbal sources, suggesting that plant-based products might harbor a richer array of *Agr-QS* inhibitors.

Moreover, synthetic simplified AIP mimics, such as Bnc3—optimized from an AIP mimic (n7OFF) by Vasquez et al.—exhibit sub-nanomolar inhibitory activity, effectively preventing natural AIP signal binding by competitively binding to *AgrC*, thus inhibiting QS signaling and reducing biofilm formation and virulence factor release [124]. These mimics can be integrated with microparticulate materials for a sustained-release effect, prolonging infection suppression. West et al. (2022) demonstrated that modified AIP mimics, trAIP-IIID2A and trAIP-IIID2Aamide, when embedded in pseudospherical PLG particles, maintained high inhibitor concentrations locally for an extended period, significantly enhancing therapeutic outcomes with reduced active agent use [125].

However, QS inhibition has also been linked to increased biofilm formation in vitro, potentially due to the suppression of phenol-soluble modulins (PSM) and protease biosynthesis regulated by the Agr system. While this inhibition affects biofilm maturation, it may also promote detachment of bacterial cells from the biofilm, potentially facilitating infection spread [126]. Thus, the use of QS inhibitors could inadvertently enhance biofilm formation by *S. aureus* at other sites, raising concerns about their impact on chronic infections.

Furthermore, the animal models employed to test Agr inhibitors predominantly involve moderate to severe skin infection scenarios, which generally do not require alternative therapeutics as these infections are often manageable without antibiotics. In contrast, severe invasive infections like sepsis or pneumonia, where Agr inhibitors could be crucial, have seldom been explored with these inhibitors, and such studies frequently exhibit significant design flaws, questioning the reliability and quorum-quenching dependence of the reported effects [127]. The potential of targeting Agr in severe systemic infections remains a critical and unresolved challenge, necessitating further investigation.

### 4.6 Phage therapy

Phage therapy (also known as bacteriophage therapy) is a promising strategy for treating biofilm-associated infections. The mechanism of action of phages differs from that of antibiotics. Phages exhibit strict host specificity and possess strong biofilm-disrupting abilities. Although phage therapy has often been overlooked in clinical practice, it is gaining renewed attention due to the rising problem of antibiotic resistance, especially in pathogens such as *S*. *aureus* [128].

In the treatment of *S*. *aureus* biofilms, phage therapy offers unique advantages through multiple mechanisms of action. For example, phages can penetrate the porous structure of biofilms and directly target bacteria hidden within them [129]. This ability enables phages to weaken biofilms effectively, promoting bacterial lysis and biofilm disruption. Furthermore, phages have been shown to carry or produce enzymes, such as polysaccharide hydrolases and DNases, which degrade the biofilm matrix. These enzymes are released during phage infection, further enhancing biofilm destruction [130]. Phages also mediate bacterial suppression through bacterial-specific genetic changes, particularly in genes related to translocation, energy metabolism, and protein synthesis. This leads to the downregulation of genes associated with bacterial energy status and ribosomal proteins [131]. In addition, phages can produce phagelysins (e.g., endolysins) that hydrolyze the peptidoglycan in bacterial cell walls. In biofilms, these endolysins can lyse individual staphylococcal cells embedded within the EPS, leading to biofilm destabilization and dissociation. This allows phages to penetrate the biofilm and target cells with low metabolic activity or in a persister state [132].

A 2024 study by Liu et al. demonstrated that the phage endolysin *LysSYL* exhibited broad-spectrum bactericidal activity against various staphylococcal strains, including methicillin-resistant *S*. *aureus* (MRSA). The study showed that *LysSYL* rapidly cleaved MRSA USA300 within 10 minutes, causing perforation and deformation of bacterial cells within the biofilm [133]. Moreover, LysSYL displayed strong eradication activity against both mono-species and mixed-species biofilms associated with *S. aureus*. This suggests that LysSYL could become an effective therapeutic agent against multi-species biofilms, and future research should focus on this potential.

As with other antimicrobial strategies, the combination of phages and antibiotics has been extensively studied. Research indicates that such therapies can significantly enhance antimicrobial effects while reducing the necessary antibiotic dosage. In a 2020 study, Kolenda et al. demonstrated that three phages (PP1493, PP1815, PP1957) displayed significant dose-dependent bactericidal activity against *S*. *aureus* embedded in biofilms in an in vitro model, both alone and in combination with antibiotics (vancomycin and rifampicin) [134]. The study also revealed a synergistic effect when phages were combined with low concentrations of antibiotics, significantly enhancing bacterial eradication in biofilms. However, no notable bactericidal effects were observed against internalized *S*. *aureus* in osteoblasts [134]. These findings underscore the potential of phages as adjuvant therapies in managing biofilm infections caused by *S*. *aureus*, although limitations persist in addressing intracellular infections. Additionally, the combination of phagelysins and antibiotics demonstrated significant biofilm-disrupting capabilities in animal models. CF-301, a purified cell wall hydrolase with strong antimicrobial activity, exhibited significant antibacterial effects when combined with daptomycin in an MRSA rat model [135]. Similarly, CF-296, a recombinant cell wall hydrolase derived from CF-301, showed enhanced anti-MRSA biofilm and antimicrobial activities in combination with daptomycin compared to monotherapy [136]. *LysSYL*, studied by Liu et al., also displayed improved effects when paired with vancomycin [133].

The promise of phage therapy lies in its adaptability and customizability. Using genetic engineering techniques, researchers can design and modify phages for greater infectivity, a broader bacterial target spectrum, and enhanced stability. Recent developments include chimeric phages and phage cocktails designed to target multiple bacterial strains or various components of biofilms simultaneously, thus improving therapeutic efficacy [137], [138], [139], [140].

Despite its advantages, the clinical application of phage therapy faces several challenges. The high specificity of phages necessitates precise identification and matching of the pathogenic bacteria infecting patients, potentially complicating diagnosis and treatment in clinical settings. Moreover, the stability and immunogenicity of phages in vivo must be optimized to prevent immune responses in patients, which could diminish treatment effectiveness. Consequently, current research is directed towards elucidating phage biology complexities, optimizing phage formulations, and conducting preclinical and clinical trials. Notably, the development of phage mixtures targeting broad-spectrum strains is a current strategy aimed at overcoming bacterial resistance and enhancing therapeutic outcomes [138].

## 5 Summary and outlook

Recent years have seen substantial progress in the study of *S. aureus* biofilms. Significant advances have been achieved in understanding the structure and formation mechanisms of biofilms, as well as their antibiotic resistance. Researchers have developed various innovative therapeutic strategies, such as enzyme therapy, nanotechnology, gene editing, and smart drug delivery systems. These strategies have enhanced the success rate of anti-infection therapies by disrupting the biofilm’s physical barrier, improving antibiotic permeability, or directly targeting biofilm-associated genes. Additionally, phage therapy and metabolic activation strategies have demonstrated potential in overcoming drug resistance in persister cells, further advancing the field of anti-biofilm therapy.

However, current research still presents certain shortcomings. Although emerging anti-biofilm strategies have shown promising results in laboratory settings, they encounter significant challenges in clinical applications, primarily due to the heterogeneity of biofilms and the drug resistance of persister cells. This heterogeneity poses substantial challenges for antibiofilm strategies to effectively target the diverse characteristics of biofilms in complex clinical environments. Moreover, the resistance exhibited by persister cells within biofilms complicates the efficacy of clinical treatments, representing a major barrier to the complete eradication of infections. Additionally, multi-species biofilms associated with *S*. *aureus* remain underexplored by these innovative strategies. This oversight may stem from the more complex antimicrobial resistance mechanisms in multi-species biofilms, where single antimicrobial strategies prove insufficient for effective eradication. Furthermore, the lack of well-established in vivo models for multi-species biofilms limits the translation of existing research findings into clinical applications.

In light of existing challenges, future research could concentrate on the creation of innovative antimicrobial substances that operate through synergistic multi-mechanistic actions. These substances ought to have the capability to simultaneously address various aspects of biofilms, potentially presenting a potent method for markedly diminishing antibiotic resistance in complex biofilms associated with *S*. *aureus*. Additionally, the precise delivery and prolonged effectiveness of drugs emerge as viable avenues for future investigation. Consequently, enhancing gene-targeting technologies and refining drug delivery mechanisms may furnish a more effective solution for antimicrobial interventions. With meticulous refinement, these methods are expected to yield more consistent and robust results in subsequent clinical trials. Nonetheless, it remains crucial to underscore that rigorous clinical research is imperative to ascertain the effectiveness of these cutting-edge strategies in targeting biofilms.

## Author statement

This manuscript has not been published previously and is not being considered for publication elsewhere. All authors have contributed significantly to the work presented and have read and approved the manuscript. No writing assistance was used in the preparation of this manuscript. None of the authors report any conflict of interest.

## Compliance with ethics requirements

This article does not involve any studies with human or animal subjects.

## CRediT authorship contribution statement

**Chen Qi:** Writing – original draft, Data curation, Conceptualization. **Jin Qiyuan:** Writing – review & editing, Writing – original draft, Formal analysis, Conceptualization. **Qiang Rui:** Writing – review & editing. **Zhao Chenhao:** Writing – review & editing. **Li Liubing:** Writing – review & editing, Writing – original draft, Funding acquisition, Formal analysis, Conceptualization. **Xie Zonggang:** Writing – review & editing. **Zhang Haifang:** Writing – review & editing, Writing – original draft, Visualization, Validation, Supervision, Investigation, Funding acquisition, Formal analysis, Data curation, Conceptualization. **Xia Yanze:** Writing – review & editing, Writing – original draft, Formal analysis, Data curation, Conceptualization. **Hu Zhenghui:** Writing – review & editing, Writing – original draft, Data curation, Conceptualization.

## Declaration of Competing Interest

The authors declare that they have no known competing financial interests or personal relationships that could have appeared to influence the work reported in this paper.
